# Integrating community pharmacy into community based anti-retroviral therapy program: A pilot implementation in Abuja, Nigeria

**DOI:** 10.1371/journal.pone.0190286

**Published:** 2018-01-10

**Authors:** Yohanna Kambai Avong, Gambo Gumel Aliyu, Bolajoko Jatau, Ritmwa Gurumnaan, Nanfwang Danat, Gbenga Ayodele Kayode, Victor Adekanmbi, Patrick Dakum

**Affiliations:** 1 Institute of Human Virology, Nigeria, Maina Court, Herbert Macaulay Way, Central Business District, Abuja, Nigeria; 2 University of Maryland School of Medicine, Baltimore, Maryland, United States of America; 3 Division of Population Medicine, School of Medicine, Cardiff University, Cardiff, United Kingdom; Faculty of Pharmacy, University of Lisbon, PORTUGAL

## Abstract

**Background:**

The landscape of Human Immunodeficiency Virus (HIV) epidemic control is shifting with the United Nations Programme on HIV/AIDS (UNAIDS) 90-90-90 benchmarks for epidemic control. Community-based Antiretroviral Therapy (CART) models have improved treatment uptake and demonstrated good clinical outcomes. We assessed the feasibility of integrating community pharmacy as a task shift structure for differentiated community ART in Abuja-Nigeria.

**Methods:**

Stable patients on first line ART regimens from public health facilities were referred to community pharmacies in different locations within the Federal Capital Territory, Abuja for prescription refills and treatment maintenance. Bio-demographic and clinical data were collected from February 25, 2016 to May 31^st^, 2017 and descriptive statistics analysis applied. The outcomes of measure were prescription refill and patient retention in care at the community pharmacy.

**Results:**

Almost 10% of stable patients on treatment were successfully devolved from eight health facilities to ten community pharmacies. Median age of the participants was 35 years [interquartile range (IQR); 30, 41] with married women in the majority. Prescription refill was 100% and almost all the participants (99.3%) were retained in care after they were devolved to the community pharmacies. Only one participant was lost-to-follow-up as a result of death.

**Conclusion:**

Excellent prescription refill and high retention in care with very low loss-to-follow-up were associated with the community pharmacy model. The use of community pharmacy for community ART is feasible in Nigeria. We recommend the scale up of the model in all the 36 states of Nigeria.

## Introduction

The success of anti-retroviral therapy (ART) in reducing the mortality, morbidity and transmission of Human Immunodeficiency Virus (HIV) has provided hope for ending the HIV epidemic in the absence of a preventive vaccine [1–4]. The World Health Organization (WHO) is promoting the use of community based ART in a global strategy to end HIV/AIDS by 2030. The community based ART approach is overwhelmingly supported because it seems to be the only viable strategy for delivering HIV treatment services closer to the people and improving ART uptake, retention in care and decongesting overburdened public health facilities [5]. In sub-Saharan Africa, the community based ART is being promoted as the key for treating the over 20 million people living with the HIV/AIDS (PLWHA) [6]. The last decade particularly witnessed a progressive shift from hospital-based ART to primary health centers and more recently to the community [7–10]. In effect, the community based approach to treating HIV/IDS has always been in the background but was not well publicized.

Different models of community ART are being implemented in sub-Sahara Africa but there is limited data on the involvement of community pharmacy in the delivery of ART within the community [11–14]. In the western countries however, several studies have documented the different roles community pharmacies can play in the treatment of HIV/AIDS [15–19].

Community pharmacies in Nigeria are registered private pharmaceutical premises licensed by the Pharmacists Council of Nigeria—the body responsible for the education of pharmacists and pharmacy practice in Nigeria. In 2014, there were 530 registered community pharmacies in the Federal Capital Territory (personal communication with Pharmacists Council of Nigeria). The community pharmacies are different from the Patent Medicine Vendors (PMV) in several ways. PMVs are registered to dispense over-the-counter drugs but the community pharmacies dispense prescription drugs and are managed by superintendent pharmacists referred here as community pharmacists (CPs). Annual license to “import, export, prepare, dispense, and distribute drugs and poisons” are issued to the CPs by the Pharmacists Council of Nigeria [20], while PMVs are issued with the PMV license.

Nigeria had the second highest burden of HIV/AIDS in the world with an estimated 3,391,546 PLWHA in 2014 [4]. Although new infections have declined from an estimated 316,733 in 2003 to 239,155 in 2014, a total of 174,253 people died from AIDS related causes in 2014 [4]. The estimated number of persons in need of ART using the eligibility criteria of CD4 of ≤350 cells/mm^3^ in 2014 was 1,665,403 (1,454,565 adults and 210,838 children) but only 748, 846 adults and children, representing 44.3% received prescription for ART in 2014 [4]. This implies that the verse majority of Nigerians living with the HIV are yet to receive ART. The “Test and Start” treatment strategy currently implemented in high burden priority local government areas will require (upon scale up) that all the estimated 3.5 million PLWHA in Nigeria to receive prescription for ART. Given that the limited health facilities in the country lack the capacity to absorb the large number of patients waiting for ART, the United States Emergency Plan for AIDS Relief (PEPFAR) program in Nigeria is planning to increase uptake of ART using the differentiated care model, which include the community pharmacy ART programme [21].

This paper presents the outcome of a pilot use of community pharmacies for ART delivery to assess the feasibility of implementation in Nigeria. There were two main objectives: (a). determine the rate of prescription refill and (b) retention in care/loss-to-follow-up over a period of 12 months.

## Methods

### Ethics

Ethical approval was given by the National Health Research Ethics Committee of Nigeria under the title: “Engaging indigenous organization to sustain and enhance clinical services for the prevention, care and treatment of HIV/AIDS in the Federal Republic of Nigeria under the President’s Emergency Plan for AIDS Relief (PEPFAR)”; Number: NHREC/01/01/2007; dated August 12, 2016. The Institute of Human Virology Nigeria, headed by Dr. Patrick Dakum, obtained this ethical clearance for all the studies conducted under the institute.

### Setting

The Federal Capital Territory houses Abuja, the capital city of Nigeria. It has a population of less than two million people and most of the urban dwellers are civil servants, while the rural areas are inhabited by farmers. The territory has six area councils: Bwari, Abuja Municipal, Abaji, Kuje, Gwagwalada and Kwali. This pilot study was conducted in the Abuja Municipal and Bwari Area Councils. These two councils are designated by the Government of Nigeria as HIV service priority Local Government Areas because of their high HIV burden. The HIV prevalence in the Abuja Municipal Area Council is estimated at 6.3% and 10.2% in the Bwari Area Council [4].

### Selection of health facilities and community pharmacies

We selected community pharmacies based on their expression of interest to participate and meeting the eligibility criteria. Prior to the selection, we approached community pharmacy owners at their professional association meetings and informed them of the study. Those who indicated interest and met the eligibility criteria of having a secured place for patient counselling, a temperature-regulated drug store, an efficient record keeping system and a superintendent pharmacist were requested to complete an evaluation form and provide a written informed consent. Priority was given to pharmacies located in areas that are readily accessible.

Eight busy health facilities were purposively selected for patient referrals. In selecting the health facilities, we considered from a list of health facilities supported by the Institute of Human Virology Nigeria through the United States President Emergency Plan for AIDS Relief project. The locations, HIV care services and willingness to provide written informed consent to participate in the pilot referral program were prioritized.

### Training of medical doctors and pharmacists

The health care providers including medical doctors and pharmacists from the selected health facilities and community pharmacies received trainings for the implementation of this pilot project. The training focused on patient recruitment, referrals, ART and pharmacovigilance and documentation. We also developed protocols for the referring facilities and community pharmacies and provided tools like laptop computers and all the official documents for record documentation. A memorandum of understanding (MoU) was also developed, which the pharmacists signed with the IHVN. The MoU is an official contract that describes the scope of work and the financial stipend the community pharmacies were paid to cover the services they provided to the participants. Community pharmacies were not permitted to charge a service-fee or charge for the drugs, i.e., patients received drugs and services free of all financial charges.

### Implementation

#### Referral system

The selected public health facilities (hospitals) and the community pharmacies were consecutively engaged in the program at different times for logistical reasons. Patient inclusion criteria into the community ART were:

1. Patient stability, defined by duration of facility-based ART of ≥ 6months with successful suppression of viral load below detection level (20 copies/ml);
2. ART regimen—only patients on first line ART regimens (Tenofovir 300mg/Lamivudine 300mg/Efavirenz600mg or Zidovudine300mg/Lamivudine150mg/Nevirapine200mg) were considered for referral to the community pharmacies
3. Willingness to participate, expressed through written consent.

Patients previously lost to follow up or had unstructured treatment interruptions were excluded from enrollment into the pilot. Pediatric and patients with co-morbidities were also excluded from enrollment. Eligible patients were given the choice to continue to receive ART from a list of ten community pharmacies located in different places within the Abuja Municipal and Bwari Area Councils.

Before the referrals, patients were informed about the benefits of community ART by trained health facility staff. Some of the benefits enumerated to the patients were lower transport cost to collect medications and better access to drug refill. They were also informed of the following risks: disclosure of HIV status from accidental breach of confidentiality and missing hospital visit if they forget to visit the health facility for viral load monitoring on the due date.

The community pharmacies were informed of patients’ referral by the health facilities ahead of time. Referral documents were prepared by the health facilities; patients took the referral document and ART prescription form (Pharmacy Order Form) to their preferred community pharmacies. The Pharmacy order form specifies the ART regimen the patient was treated with prior to the referral; the CPs dispenses this original regimen in bimonthly prescription refill visits. In some health facilities, patients were referred with about two to three months’ worth of drugs, while in other health facilities; drugs were not given at the time of referral. For patients who were referred with drugs, visit to the community pharmacies for prescription refill was delayed until the drugs were almost completely exhausted. For the patients who were referred without drugs, visit to the community pharmacies to refill prescription was done almost immediately.

#### ART refills

ART prescriptions written by physicians were dispensed by the CPs who also provided counseling and monitored adherence and adverse drug reactions in addition to drug logistic and data management services. Prescriptions were generally refilled bimonthly but in special circumstances (like when a patient has to travel away from his home for many months), a three month worth of medications could be dispensed. Only adult first line regimens and Cotrimoxazole prophylaxes are offered but there is plan to expand the regimen mix to include second line regimens.

During the pilot phase, drug dispensing took place in secured locations to protect patients’ privacy and confidentiality while counseling. The CPs collected routine care information and captured the data in patient’s treatment card and program computer software called “Intelligent Dispensing of Antiretroviral Treatment” (iDART). The next refill appointment date was given to the patient at the end of each refill activity. Patients observed to be ill or in need of clinical care or those with adverse drug reaction were referred back to the health facilities that referred them to the community pharmacy for evaluation and treatment. Patients remained permanently linked to the health facilities through such referrals and routine care assessment like the viral load tests. The CPs were responsible for ensuring patients returned to the hospitals for routine care assessment at the due date. They also provided leadership and management services in maintaining cordial relationships with patients and other health care providers. Studies have shown that optimal adherence and retention in care are driven by healthcare providers’ attitude to the patients in addition to factors like counselling, peer support group, reduced pill burden and short travel distance to health facility [22–26].

#### Pharmacovigilance services

Pharmacovigilance service was included in our model because several studies in the United States of America, Europe and Brazil have reported cardio-metabolic disorders associated with the use of antiretroviral drugs [27–30]. These disorders have also been reported in sub-Sahara Africa among people treated for HIV [31–36]. Monitoring and reporting of ADRs was therefore considered an important activity for improving the quality of care for the community pharmacy ART model.

### Data collection

Program data for this pilot project were accessed through lap top computers provided to the participating community pharmacies and referring hospitals. These data were transferred online to a central server on weekly basis. S1 Table. Access to the database is protected by two level passwords at the initial log on and for the database.

De-identified data were abstracted and organized into cell counts in which descriptive values were provided along with frequencies and proportions. We collected data from February 25, 2016 to May 31^st^, 2017 and applied descriptive statistics to report our findings.

## Results

A total of 295 participants were consecutively referred from eight public health facilities to ten community pharmacies within the 12 months review period. Median age was 35 years (30; 41); married women constituted 72% and the “self-employed” (e.g., business men, artisans) participants accounted for 42.8%. Baseline median viral load was 19 copies/mL and CD4 counts, 460 cells/mL (Table 1).

**Table 1:** Baseline demographic and clinical characteristics of the participants.

Sociodemographic characteristics	Number (%); Median [IQR*]
**Age** (years)	35 [30; 41]
**Gender** Women Men	208 (71.0) 85 (29.0)
**Marital Status** Married Single Widow	200 (72.7) 68 (24.7) 7 (2.6)
**Employment** Self Employed Employed Unemployed	119 (42.8) 93 (33.5) 66 (23.7)
**Clinical characteristics**	
Viral load (Copies/mL) CD4 count(Cells/mL)	19 [19; 32] 460 [277; 648]

*IQR = interquartile range

Table 2 presents the outcome of the pilot within the review period. Almost 10% (295) of the people treated for HIV/AIDS at the study centers were devolved to community pharmacies. The rate of prescription refill was excellent (100%)–on the average, participants’ refilled prescriptions bimonthly four times, which is equivalent to eight months of continuous therapy in the community pharmacies. Retention in care was very high (99.3%) and only one participant was lost-to-follow -up.

**Table 2:** Overall outcome of the pilot study within the review period.

Assessment variable	Number (%); Median [IQR]
Participants referred to community pharmacies	295 (8.3%)
Median prescription refill*	4 [2; 5]
Prescription refill rate	100%
Median Follow up period (months)	8 [4;10)
Retention in care (in the community pharmacy)	287 (99.3%
Lost-to-follow-up	1 (0.35%)
Death	1 (0.35%)

Note *: The median prescription refill is a measure of the number of times participants’ refilled prescriptions.

## Discussion

This Pilot Community-based ART delivery Model has demonstrated the feasibility of using the community pharmacies in Nigeria for community HIV management. Previous studies reported the successes of other community based ART models [7,9–11], however, some of these models are not based on sustainable structures within the community. Our model is based on the community pharmacies, which are existing structures the communities patronize for health care services.

There are three important findings that our model has demonstrated. Firstly, we showed that patients referral from the health care facilities to the community pharmacies is feasible and sustainable. Secondly, excellent prescription refill and retention in care—significant variables for ending HIV, can be achieved and thirdly, we witnessed a gradual decongestion of health facilities through the referral activities. The decongestion has the advantage of creating more space for new patients to be recruited and at the same time, freeing health care providers’ time for the treatment of critical and complicated co-morbidities. Our findings support what other studies have reported on the potential of community based ART to improve retention in care and decongest over-crowded public hospitals [7,10,11].

A major strength of our model is its foundation on the community pharmacy, recognized as the first point of call for patients seeking health service in the community. But there are several limitations that may constrain the full operationalization of the model. The community pharmacies do not exist in some remote rural communities where only the Patent Medicine Vendors operate. However, most of the people living with HIV/AIDS live in the sub-urban areas [37] where the community pharmacies can be found. Another significant limitation of the model is the overwhelming support it enjoyed; the community pharmacies would probably not have performed as they did if they were not well resourced. However, despite these limitations, the community pharmacy model can become an overarching comprehensive HIV care model that would play a central part in the overall response to the HIV epidemic in Nigeria, if rolled out in every part of the country. The model can also promote the realization of the “treat all” global strategy for ending HIV, considering that most of the people living with HIV/AIDS are in the communities where the model can be operated.

### Public health significance of the model

In Africa, qualified clinical staffs to provide optimal care, especially ART is lacking [38]. In rural Africa, the low geographical density of health structures creates a heavy burden on patients who may have to travel long distances to seek care. A study conducted in North-Central Nigeria by Avong et al [39] found that patients paid a median of USD5:00 (equivalent to 2,000 Nigerian Naira) to travel to the study centre to access ART. In the urban settings, competing activities such as work and social life interfere with time spent queuing in overburdened health facilities with large patient cohorts. Long waiting time is associated with high attrition among patients on ART [40]. These challenges can largely be offset by the community-based ART. In Uganda, a home-based ART delivery model showed similar survival and virological suppression with a facility-based ART [11]. In Western Kenya, community-based care provided by PLWHA resulted in similar clinical outcomes as facility care with half the number of clinic visits [12,13]. In Tanzania, a model of ART delivery by community-based volunteers linked to trained medical staffs led to fewer patients being lost to follow-up from treatment [14]. In Mozambique, a rotation community support approach was applied and more than 90% retention at 4 years was reported [41,42]. In Nigeria, our study has demonstrated the feasibility of sustaining high retention in care and low loss-to-follow–up. Some authors have suggested that the community based ART is the key for lifelong ART for the 20 million people in sub-Sahara Africa [6]. But opponents of the community based interventions argue that once patients stop coming to the hospital to see the physicians, clinical care and its accrued benefits will depreciate leading to poor retention in care and loss-to-follow-up. This argument has however been disproved by several studies that demonstrated comparable clinical and virologic benefits between facility based and home-based treatments [12,14]. This pilot survey has provided additional evidence in support of the community based ART approach. With task shifting of HIV care for stabilized patient to CPs and increased uptake of ART services, the spread of HIV can be minimized with sustained viral suppression and contributing to fast tracking the UNAIDS 90:90:90 strategy.

## Conclusion

Use of community pharmacies for HIV treatment maintenance is feasible in Nigeria with potential signs of good retention in care. Community pharmacies have a role to play in providing community ART services and reducing burden on patients and health care providers. Longitudinal studies (cohort and quasi-experimental) are however needed to provide evidence of cost-effectiveness to inform future large scale implementation.

## Supporting information

S1 Table Sample of treatment data transferred to the central server. (XLSX)
